# Prostate surgery outcomes in patients presenting with and without a urethral catheter: a long-term comparative study

**DOI:** 10.1007/s00345-026-06702-9

**Published:** 2026-09-04

**Authors:** Niv Segal, Yaron Ehrlich, David Lifshitz, Abd E. Darawsha

**Affiliations:** Department of Urology, Rabin Medical Center - Hasharon Hospital, Petach Tikva, Israel

**Keywords:** Benign prostatic enlargement, Indwelling urinary catheter, Urinary retention, Prostate surgery outcomes, Lower urinary tract symptoms, Long-term outcomes

## Abstract

**Purpose:**

To evaluate long-term catheter-free spontaneous voiding after surgery for benign prostatic enlargement (BPE) among patients presenting with an indwelling urethral catheter (IUC), using age-matched patients undergoing surgery for lower urinary tract symptoms (LUTS) without a catheter as a clinical reference group.

**Methods:**

We retrospectively analyzed 481 patients treated for BPE at our center between January 2017 and March 2019 using open or endoscopic surgical techniques. Of 143 patients referred with an IUC, 143 age-matched patients operated on for LUTS alone were selected as a clinical reference group (total *n* = 286). Telephone follow-up was performed in late 2021 after a median of 2.5 years (IQR 637–1140 days).

**Results:**

Patients with IUC had larger prostates, higher preoperative post-void residual volumes, and more positive urine cultures than patients with LUTS alone. Early postoperative retention was numerically more frequent in the IUC group, while long-term catheter dependence was 4% versus 2%.

**Conclusion:**

Despite worse baseline urologic characteristics, most patients presenting with a preoperative indwelling urethral catheter achieved durable catheter-free spontaneous voiding at long-term follow-up after BPE surgery. Surgery may therefore be a reasonable option for appropriately selected catheterized patients.

**Supplementary Information:**

The online version contains supplementary material available at https://doi.org/10.1007/s00345-026-06702-9.

## Introduction

Patients with acute urinary retention are typically managed initially with an indwelling catheter, often followed by one or two trials of catheter withdrawal [1] Patients with high post-void residual are commonly treated with either clean intermittent catheterization (CIC) or an indwelling catheter [2].

Patients presenting with an IUC before prostate surgery may experience less favorable outcomes. Pickard et al. [3] reported that 9.2% of men with urinary retention were unable to void after surgery and required postoperative catheterization, which became permanent in 0.9% of cases. In contrast, among patients without preoperative retention, the corresponding rates were 2.3% and 0.1%, respectively. In an often-cited study, Mebust et al. [4] reported an 11% failure-to-void rate among patients with urinary retention undergoing TURP. In a population-based study, Chen et al. reported a re-catheterization rate of 13.8% among 3,305 patients undergoing TURP who presented with an IUC, compared with 0% among 1,062 patients presenting without a catheter [5].

A subset of patients referred for surgery undergoes urodynamic testing, particularly those presenting with an IUC. Among these patients, some are suspected of having an element of detrusor underactivity [6] It is well recognized that not all cases of urinary retention are attributable solely to mechanical obstruction; up to 40% of patients may exhibit some degree of detrusor underactivity [6] The EAU Guidelines on Non-Neurogenic Male LUTS state that pressure-flow studies are not routinely required before prostate surgery for all patients, and recommend urodynamic studies only in selected patients when specific indications exist or when further evaluation of the underlying pathophysiology is warranted [7].

The primary objective of this study was to evaluate catheter-free spontaneous voiding at long-term follow-up after BPE surgery among patients presenting with a preoperative indwelling urethral catheter. An age-matched group undergoing surgery for LUTS without a preoperative catheter was included as a clinical reference group. Secondary outcomes included early postoperative urinary retention, postoperative urinary tract infection, urinary incontinence, IPSS at long-term follow-up, and the need for an indwelling catheter or clean intermittent catheterization during follow-up.

## Methods

### Study design and patient population

We conducted a retrospective cohort study of all consecutive patients who underwent surgery for benign prostatic enlargement (BPE) at Rabin Medical Center, Hasharon Campus, between January 2017 and March 2019. No exclusion criteria were applied to the source surgical cohort. Surgical procedures included transurethral resection of the prostate (TURP), holmium laser enucleation of the prostate (HoLEP), retropubic prostatectomy (RPP), suprapubic prostatectomy (SPP), and transurethral incision of the prostate (TUIP).

During the study period, 481 patients underwent surgery for BPE. Of these, 143 patients presented for surgery with a preoperative indwelling urethral catheter (IUC) because of urinary retention. An age-matched clinical reference group comprised 143 patients who underwent surgery for lower urinary tract symptoms (LUTS) without a preoperative IUC, selected from the same institutional surgical cohort and study period and matched 1:1 by age.

### Data collection and definitions

Clinical, perioperative, and follow-up data were collected retrospectively from institutional medical records. Preoperative variables included age, prostate volume, preoperative post-void residual volume, urine culture results, history of previous prostate surgery, duration of catheterization before surgery, number of catheter-withdrawal trials before surgery, and preoperative urodynamic testing when available. Perioperative variables included type of surgery, length of admission, duration of postoperative catheterization, catheter status at discharge, residual urine volume before discharge, early postoperative urinary retention, urinary tract infection, urinary incontinence, and catheter status at early clinic follow-up.

Previous prostate surgery was defined as any prostate operation performed before the index surgery during the study period. The index surgery was defined as the BPE operation included in the present analysis.

### Urodynamic testing

Preoperative urodynamic testing was not performed routinely in all patients. It was performed selectively according to the treating physician’s clinical judgment, particularly in patients in whom impaired detrusor contractility or a non-obstructive component of urinary retention was suspected. Urodynamic findings were analyzed descriptively because only a small subset of patients underwent testing.

### Follow-up assessment

In addition to routine postoperative clinic follow-up, which was generally scheduled within 30 to 45 days after surgery, delayed telephone follow-up was performed at the end of 2021 specifically for the purpose of the study to assess long-term functional outcomes. Telephone follow-up was conducted after a median follow-up of 2.5 years. During the telephone interview, patients were asked about current voiding status, use of an indwelling catheter or clean intermittent catheterization (CIC), recurrent urinary retention, urinary tract infection since the last clinic visit, urinary incontinence, and LUTS severity using the International Prostate Symptom Score (IPSS), when available. Patients who reported concerning symptoms, including severe LUTS, recurrent urinary tract infection, orchiepididymitis, recurrent urinary retention, or ongoing need for catheterization, were invited for in-person clinical assessment. Postoperative urodynamic testing was not performed routinely after the telephone follow-up and was reserved for selected patients according to clinical judgment.

### Outcomes

The primary outcome was catheter-free spontaneous voiding at long-term follow-up, defined as absence of an indwelling catheter and no need for CIC. Secondary outcomes included early postoperative urinary retention, catheter status at discharge, catheter status at early clinic follow-up, urinary tract infection, urinary incontinence, IPSS at long-term follow-up, and recurrent urinary retention or urinary tract infection between the last clinic visit and delayed telephone follow-up.

### Ethical approval

The study was approved by the Helsinki Commission of Rabin Medical Center, Hasharon Campus (approval No. 0487-19-RMC). The requirement for written informed consent was waived by the institutional Helsinki Commission as part of the study approval. Patients who reported concerning symptoms during telephone follow-up were invited for in-person clinical evaluation.

### Statistical analysis

Statistical analyses were performed using SPSS version 26. Continuous variables were summarized as mean ± standard deviation or median with interquartile range, as appropriate, and categorical variables as frequencies and percentages. Between-group comparisons were performed using the independent-samples t test, Mann-Whitney U test, chi-square test, or Fisher’s exact test, as appropriate. Fisher’s exact test was used when expected cell counts were small. All tests were two-sided, and *p* < 0.05 was considered statistically significant. As an exploratory sensitivity analysis, propensity-score matching used age, prostate volume, preoperative post-void residual volume, urine-culture status, and previous prostate surgery, with 1:1 nearest-neighbor matching and a caliper of 0.2 standard deviations of the logit of the propensity score.

## Results

The study included 286 patients, divided into two equal groups of 143: patients with an IUC and patients operated on for LUTS without a catheter. Mean age was similar between groups (71.8 ± 8.9 vs. 71.5 ± 6.7 years, *p* = 0.72). The IUC group had significantly larger prostates (96 ± 47 g vs. 83.6 ± 37 g, *p* = 0.015), higher preoperative post-void residual volumes (1063 ± 510 mL vs. 235 ± 117 mL, *p* < 0.01), and more positive urine cultures (47% vs. 3.5%, *p* < 0.01). Median preoperative catheter duration in the IUC group was 60 days (IQR 30–90), and 47% had at least one failed catheter-withdrawal trial before surgery. Among patients in the preoperative IUC group, 112 of 138 (81.2%) presented with painful urinary retention and 26 of 138 (18.8%) with non-painful urinary retention. Baseline characteristics are shown in Table 1.

Perioperative and early postoperative outcomes are summarized in Supplementary Table 1. Patients with a preoperative IUC had a longer hospital stay and longer postoperative catheterization, and tended to have higher residual urine volumes and more early retention events. Rates of failed catheter withdrawal before discharge, catheterization at first follow-up, urinary tract infection, and incidental prostate cancer did not differ significantly between groups. Although the absolute difference in time to first follow-up between groups was modest, approximately 10 days, it was statistically significant, therefore, early postoperative comparisons should be interpreted with caution.

Late postoperative outcomes are shown in Table 2. After a median follow-up of 2.5 years, an IUC was present in 6 patients (4%) in the IUC group and 3 patients (2%) in the LUTS group (*p* = 0.71). Overall urinary incontinence was more common in the IUC group (18.9% vs. 7.7%, *p* = 0.016), No statistically significant between-group differences were observed in late urinary tract infection episodes or IPSS scores.

**Fig. 1:**
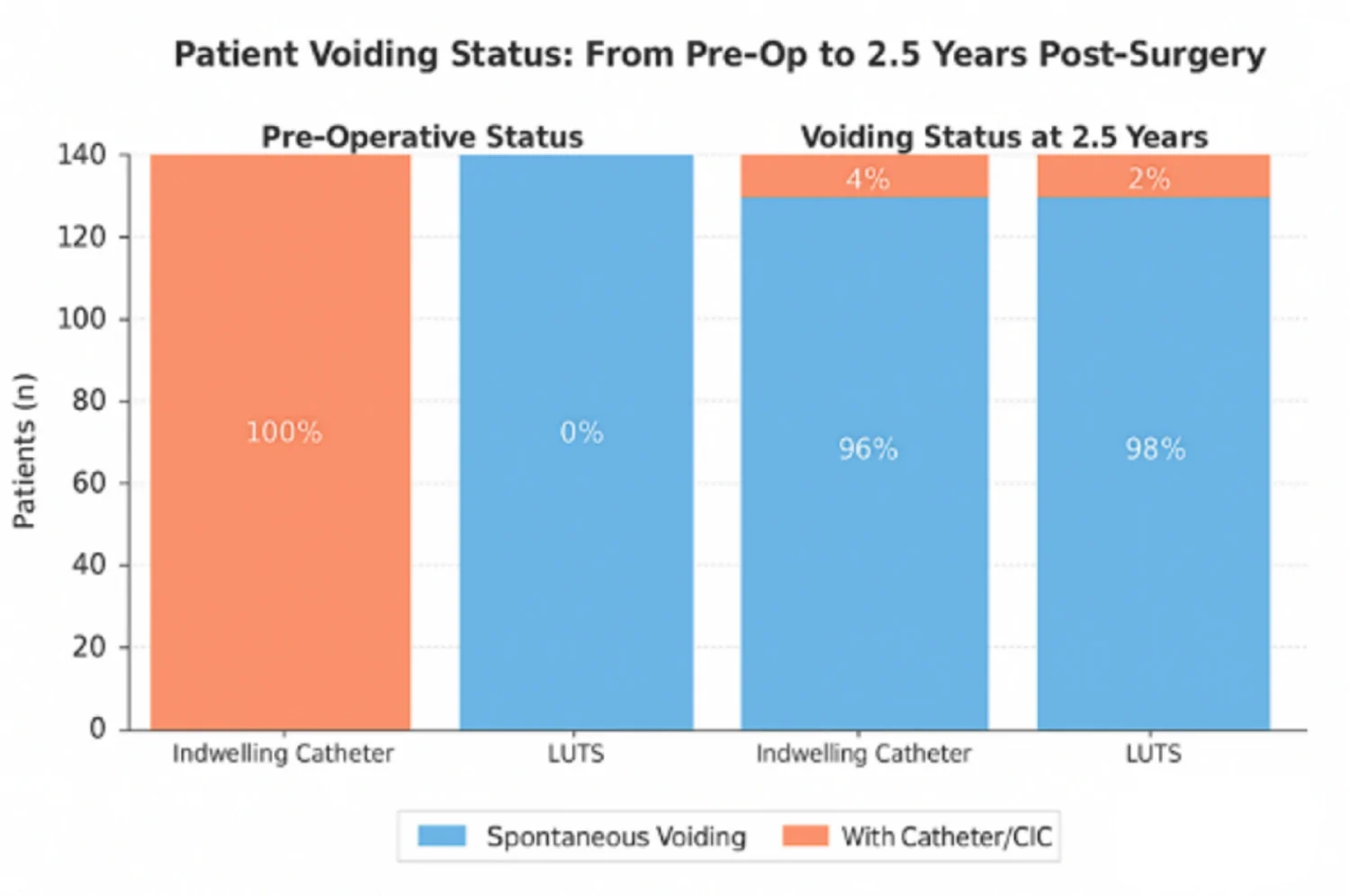
Patient voiding status from pre-op to 2.5 years post-surgery

As shown in Fig. 1, catheter-free spontaneous voiding at long-term follow-up was observed in 137 of 143 patients (95.8%) in the IUC group and 140 of 143 patients (97.9%) in the LUTS group.

Among the 10 patients who underwent urodynamic studies while catheterized at the time of surgery, 9 had detrusor underactivity and all regained spontaneous urination at long-term follow-up.

## Discussion

This study evaluated long-term postoperative outcomes after BPE surgery among patients presenting with a preoperative indwelling urethral catheter, with age-matched patients undergoing surgery for LUTS serving as a clinical reference group. The main finding was that 95.8% of patients in the catheterized group were catheter-free at long-term follow-up despite worse baseline urologic characteristics.

This finding is clinically relevant because patients presenting with an indwelling catheter are often considered to have a less favorable functional prognosis, particularly when urinary retention is longstanding or when detrusor underactivity is suspected. In the present cohort, the median duration of catheterization before surgery was 60 days, almost half of the catheterized patients had at least one failed trial without catheter before surgery, and nearly 20% presented with non-painful urinary retention, which may suggest a more chronic retention phenotype and potentially less favorable baseline bladder function. Nevertheless, most patients regained and maintained spontaneous voiding during long-term follow-up.

The two study groups were not expected to be identical at baseline. Patients presenting with a preoperative indwelling urethral catheter represent a clinically different and generally more severe urinary-retention phenotype than patients referred for surgery because of LUTS alone. Although age and ASA score were similar between groups, important urologic baseline differences remained. Accordingly, the larger residual urine volumes, higher rate of positive urine cultures, and other adverse baseline characteristics in the catheterized group likely reflect the underlying clinical presentation. Therefore, the present study should be interpreted as a comparison of long-term outcomes between two clinically distinct referral populations, rather than as a causal analysis of the isolated effect of catheter status. Exploratory propensity-score matching similarly demonstrated limited baseline overlap and could not provide a reliable adjusted estimate.

The present cohort also reflects routine clinical practice rather than a highly standardized or highly selected surgical series. Previous studies of patients undergoing surgery with an indwelling catheter often focused on selected populations, single surgical techniques, or did not report important clinical factors such as catheter duration, previous catheter removal attempts, or comorbidities that may contribute to LUTS and urinary retention, including diabetes, prior cerebrovascular events, and neurological disease [8–12]. In the present study, the median duration of catheterization before surgery was 60 days, nearly 47% of patients had at least one failed catheter removal trial before surgery, and patients with relevant comorbidities were not excluded. These features may increase the real-world applicability of the findings but also introduce clinical heterogeneity and residual confounding.

Clean intermittent catheterization may represent an alternative or bridging strategy for selected patients with urinary retention or high post-void residual volume. However, because this was a retrospective study, preoperative catheter management was not standardized. The choice between indwelling catheterization, intermittent catheterization, and timing of surgery was determined by routine clinical practice rather than by study protocol. Therefore, the potential influence of different preoperative catheter-management strategies on postoperative outcomes could not be reliably assessed.

The urodynamic findings should be interpreted with caution. Only a small subset of catheterized patients underwent preoperative urodynamic testing. Although all catheterized patients with detrusor underactivity in this subgroup regained spontaneous voiding, the number of patients was too small to support predictive conclusions, and patients selected for urodynamic evaluation may not be representative of the overall cohort. These findings should therefore be viewed as descriptive only.

This study has several limitations. First, it was retrospective and single-center in design. Second, age matching did not eliminate baseline differences, and exploratory propensity-score matching showed limited overlap, precluding a reliable adjusted comparison. Residual confounding, including by diabetes and neurologic comorbidities, therefore remains possible. Third, surgical heterogeneity and surgeon-related variability may have influenced outcomes. Fourth, only a small subset of patients underwent preoperative urodynamic testing. Finally, the low number of patients with long-term catheter dependence limited the ability to identify reliable predictors of failure to resume spontaneous voiding.

## Conclusion

Despite worse baseline urologic characteristics, most patients presenting with a preoperative indwelling urethral catheter achieved durable catheter-free spontaneous voiding at long-term follow-up after BPE surgery. Surgery may therefore be a reasonable option for appropriately selected catheterized patients.

**Table 1:** Baseline characteristics of patients with preoperative IUC and patients undergoing surgery for LUTS without preoperative IUC

Characteristic	LUTS without preoperative IUC *N* = 143	Preoperative IUC *N* = 143	*P* value
Age (years), mean (SD)	71.5 (± 6.7)	71.8 (± 8.9)	0.72
Prostate size (g), mean (SD)	83.6 (± 37)	96 (± 47)	0.015
ASA score, median (IQR)	2 (2–3)	2 (2–3)	NS
Positive urine culture, n (%)	5 (3.5%)	67 (47%)	< 0.01
Had urodynamics pre-op	15 (10.5%)	10 (7%)	NA
Post-void residual before surgery (mL), mean (SD)	235 (± 117)	1063 (± 510)	< 0.01
Urinary retention presentation in the IUC group, n (%)	NA	Painful 112/138 (81.2%)	NA
		Non-painful 26/138 (18.8%)	
Duration of IUC before surgery, days median (IQR)	NA	60 [30–90]	NA
Catheter-withdrawal trials before surgery	NA	None: 78 (53%)	NA
		Once or more: 65 (47%)	
Had a prior procedure	14 (10%)	3 (2%)	< 0.01
Type of prior procedure	11 TURP; 2 TUIP; 1 SPP	3 TURP	
Type of surgery	HoLEP 59 (41.26%)	HoLEP 53 (37.06%)	0.27
	TURP 67 (46.85%)	TURP 59 (41.26%)	
	SPP 5 (3.50%)	SPP 11 (7.69%)	
	RPP 11 (7.69%)	RPP 19 (13.29%)	
	TUIP 1 (0.70%)	TUIP 1 (0.70%)	

Values are presented as mean ± standard deviation, median [interquartile range], or n (%), as appropriate. Retention-type data were available for 138 of 143 patients in the IUC group

*IUC* indwelling urethral catheter,* LUTS* lower urinary tract symptoms,* HoLEP* holmium laser enucleation of the prostate, *TURP* transurethral resection of the prostate,* SPP* suprapubic prostatectomy,* RPP* retropubic prostatectomy,* TUIP* transurethral incision of the prostate

**Table 2:** Late postoperative characteristics at 2.5-year median follow-up

Characteristic	LUTS without preoperative IUC *N* = 143	Preoperative IUC *N* = 143	*P* value
Time to late telephone follow-up, days (median, IQR)	860 (619–1039)	812 (637–1140)	0.09
Catheter dependence at late follow-up, n(%)	3 (2%)	6 (4%)	0.71
*Any urinary incontinence, n(%)*	11 (7.7%)	27 (18.9%)	0.016
Mild SUI	6 (4.2%)	21 (14.7%)	
Moderate (pads/day)	3 (2.1%)	1 (0.7%)	
Severe (diaper)	2 (1.4%)	5 (3.5%)	
UTI episodes since last clinic visit, n(%)	8 (5.5%)	16 (11%)	0.75
IPSS score (median, IQR)	5 (5–7)	6 (5–8)	0.86

Values are presented as median [interquartile range] or n (%), as appropriate

*IUC* indwelling urethral catheter,* LUTS* lower urinary tract symptoms,* IPSS* International Prostate Symptom Score,* SUI* stress urinary incontinence,* UTI* urinary tract infection

## Supplementary Information

Below is the link to the electronic supplementary material.

Supplementary Material 1

## Data Availability

No datasets were generated or analysed during the current study.
