# Supplementary material for: Prostate surgery outcomes in patients presenting with and without a urethral catheter: a long-term comparative study
Source: World J Urol. 2026 Sep 4;44(1):640. doi: 10.1007/s00345-026-06702-9 (PMC13545057; doi:10.1007/s00345-026-06702-9)
Supplement: Supplementary file 1 [file 345_2026_6702_MOESM1_ESM.docx]

Supplementary Material

Supplementary Table 1. Perioperative and early postoperative outcomes in patients with preoperative IUC and patients undergoing surgery for LUTS without preoperative IUC

| **Characteristic** | **LUTS without preoperative IUC  N=143** | **Preoperative IUC  N=143** | **P value** |
| --- | --- | --- | --- |
| **Admission and immediate postoperative characteristics** | | | |
| Length of hospital stay, days median (IQR) | 3 (2-4) | 4 (2-6) | <0.001 |
| Duration of postoperative catheterization, days, median (IQR) | 2 (1-2) | 2 (1-4) | <0.02 |
| Failed catheter withdrawal at discharge, n (%) | 2 (1.4%) | 7 (5%) | 0.092 |
| Urinary residual before discharge, mL median (IQR) | 35 (14-64) | 56 (29-109) | <0.001 |
| **Early postoperative characteristics (first clinic visit)** | | | |
| Time to first follow-up visit, days median (IQR) | 33 (28-43) | 43 (33-117) | <0.001 |
| Urinary tract infection episodes after surgery, n (%) | 3 (2%) | 10 (7%) | 0.55 |
| Urinary retention episodes following discharge (first 30 days), n (%) | 4 (3%) | 14 (10%) | 0.085 |
| Indwelling catheter at first clinic follow-up, n (%) | 0 | 3 (2%) | 0.55 |
| Carcinoma of prostate, n (%) | 5 (3.5%) | 10 (7%) | 0.18 |

*Fisher exact test was used where applicable.*

**Supplementary Figure 1. Study flow diagram and follow-up time points**
